# Supplementary material for: Discovery of beneficial haplotypes for complex traits in maize landraces
Source: Nat Commun. 2020 Oct 2;11:4954. doi: 10.1038/s41467-020-18683-3 (PMC7532167; doi:10.1038/s41467-020-18683-3)
Supplement: Supplementary file 1 — Supplementary Information [file 41467_2020_18683_MOESM1_ESM.pdf]

Supplementary Information

Discovery of beneficial haplotypes for complex traits in maize landraces

Mayer et al.

Supplementary Figures

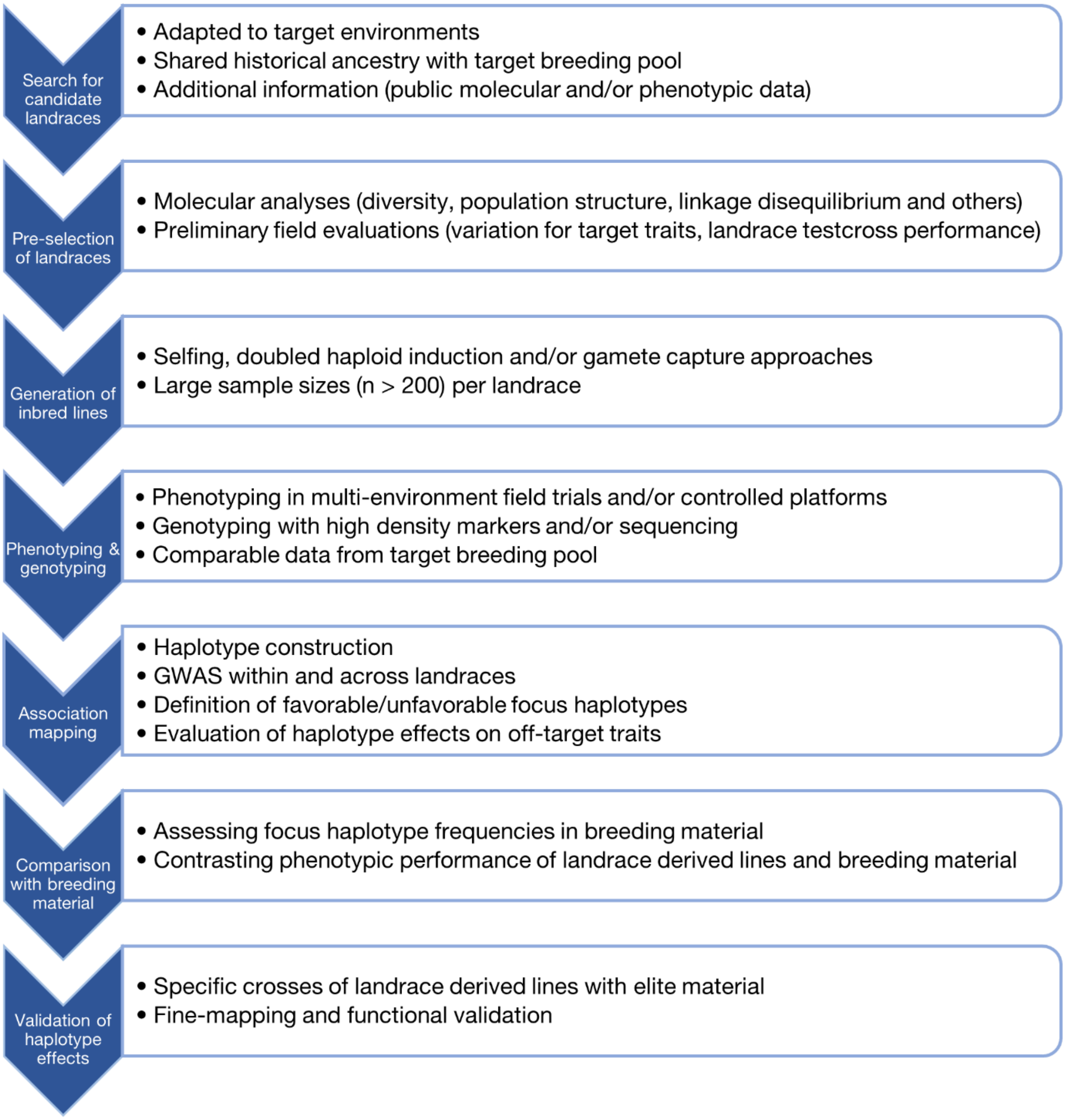

Supplementary Fig. 1: Workflow for making native diversity of landraces accessible for the improvement of elite germplasm.

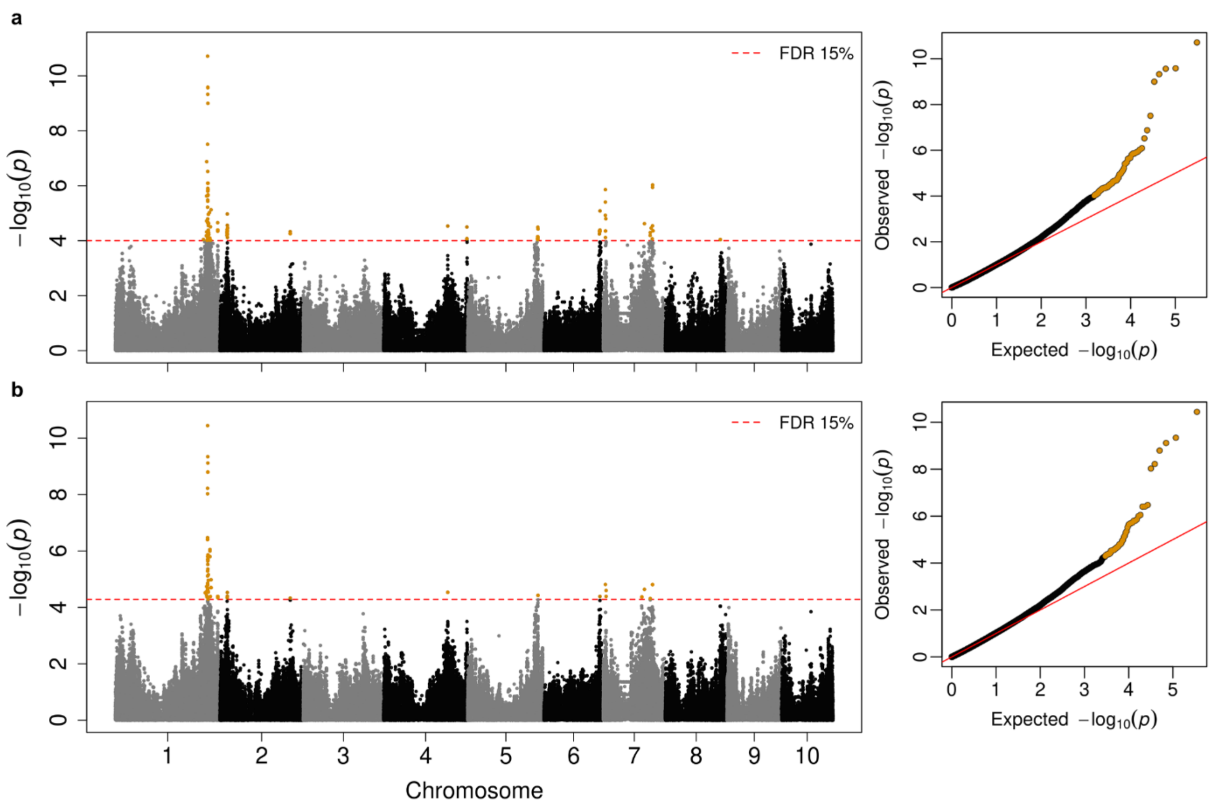

Supplementary Fig. 2: Genome-wide association scans for tillering based on haplotypes and SNPs. Manhattan plots (left) and corresponding QQ plots (right) for the **(a)** haplotype and **(b)** SNP based univariate GWAS in 899 landrace-derived DH lines, using adjusted genotype means across five tested environments. Significant associations are colored in orange. Results are based on **(a)** 154,104 haplotypes and **(b)** 175,810 SNPs, respectively. Source data are provided as a Source Data file.

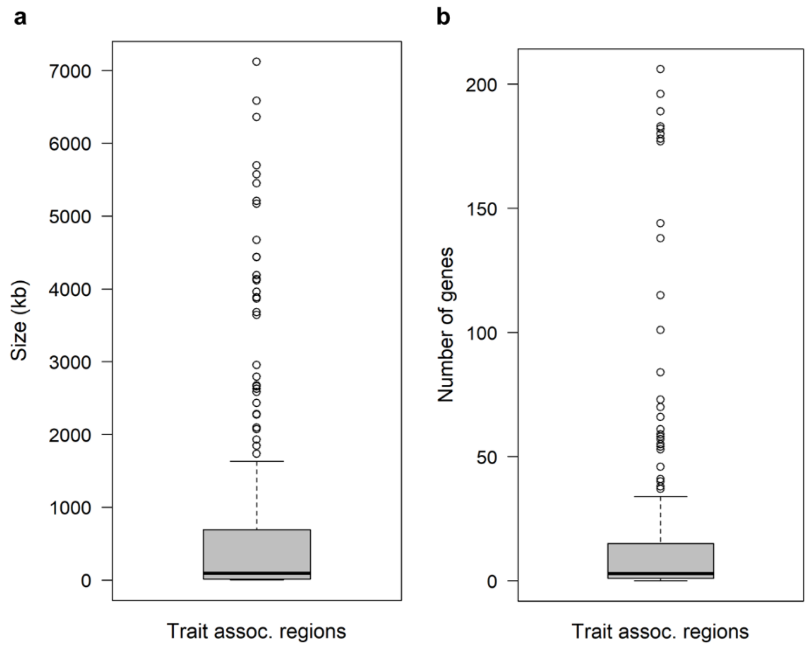

Supplementary Fig. 3: Size and number of annotated genes of/in trait-associated genomic regions. In total 324 genomic regions associated with the traits EV_V4, EV_V6, PH_V4, PH_V6, PH_final, FF, MF, LO or TILL were discovered in 899 DH lines derived from three maize landraces. Boxplots show the upper and lower quartile, median (bold horizontal bar) and whiskers (dashed vertical lines) of the **(a)** size and **(b)** number of annotated genes of/in the 324 genomic regions. Points outside the whiskers indicate values ±1.5 times the interquartile range. Source data are provided as a Source Data file.

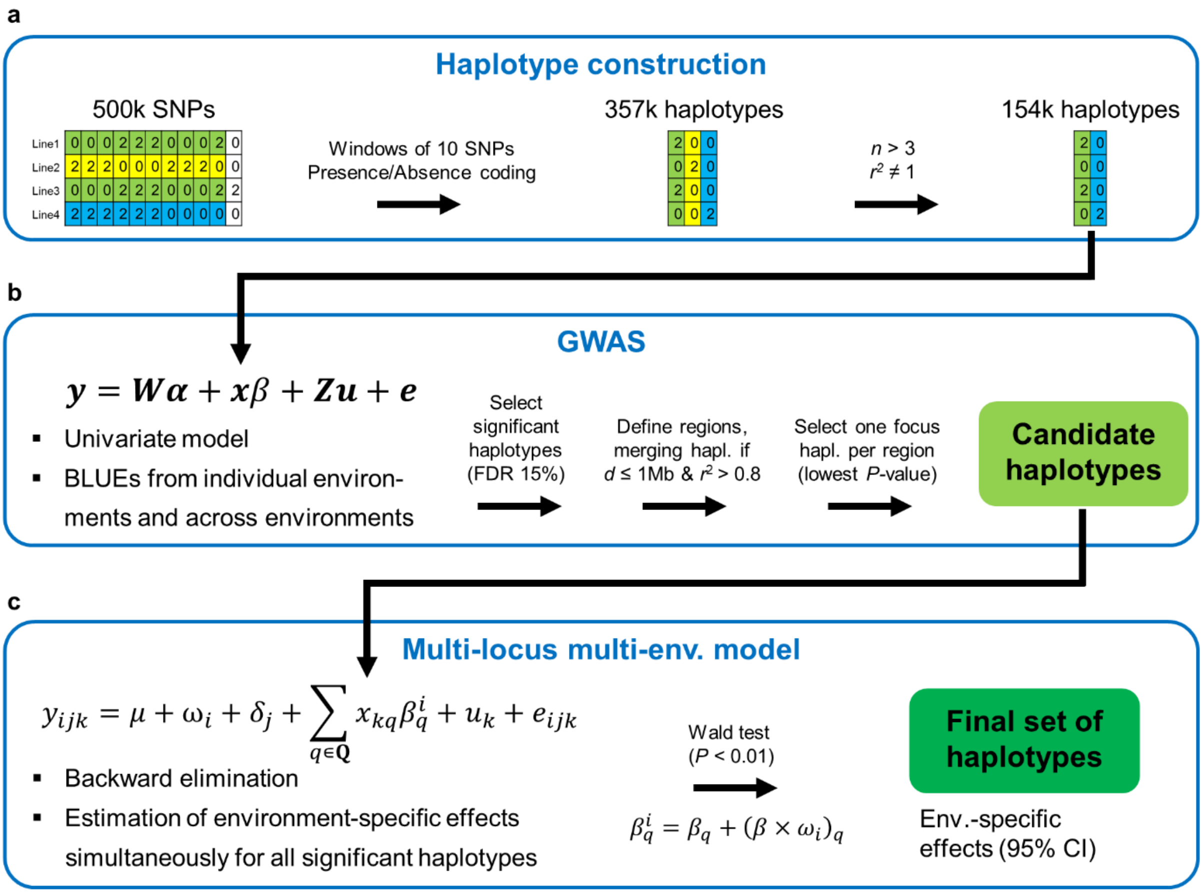

Supplementary Fig. 4: Flowchart of experimental analyses. (a) Construction of haplotypes. The DH panel comprised 357k haplotypes in total. Thereof, 154k haplotypes remained after filtering for $n > 3$ and $r^2 \neq 1$. **(b)** GWAS conducted for up to 11 single environments as well as for the across environment BLUEs for the combined set of 899 genotyped DH lines derived from three landraces. **(c)** Multi-locus, multi-environment model for performing backward elimination of candidate haplotypes (Wald test, two-sided) and estimating environment-specific haplotype effects for the final set $\boldsymbol{Q}$ of focus haplotypes. See Methods for model notations.

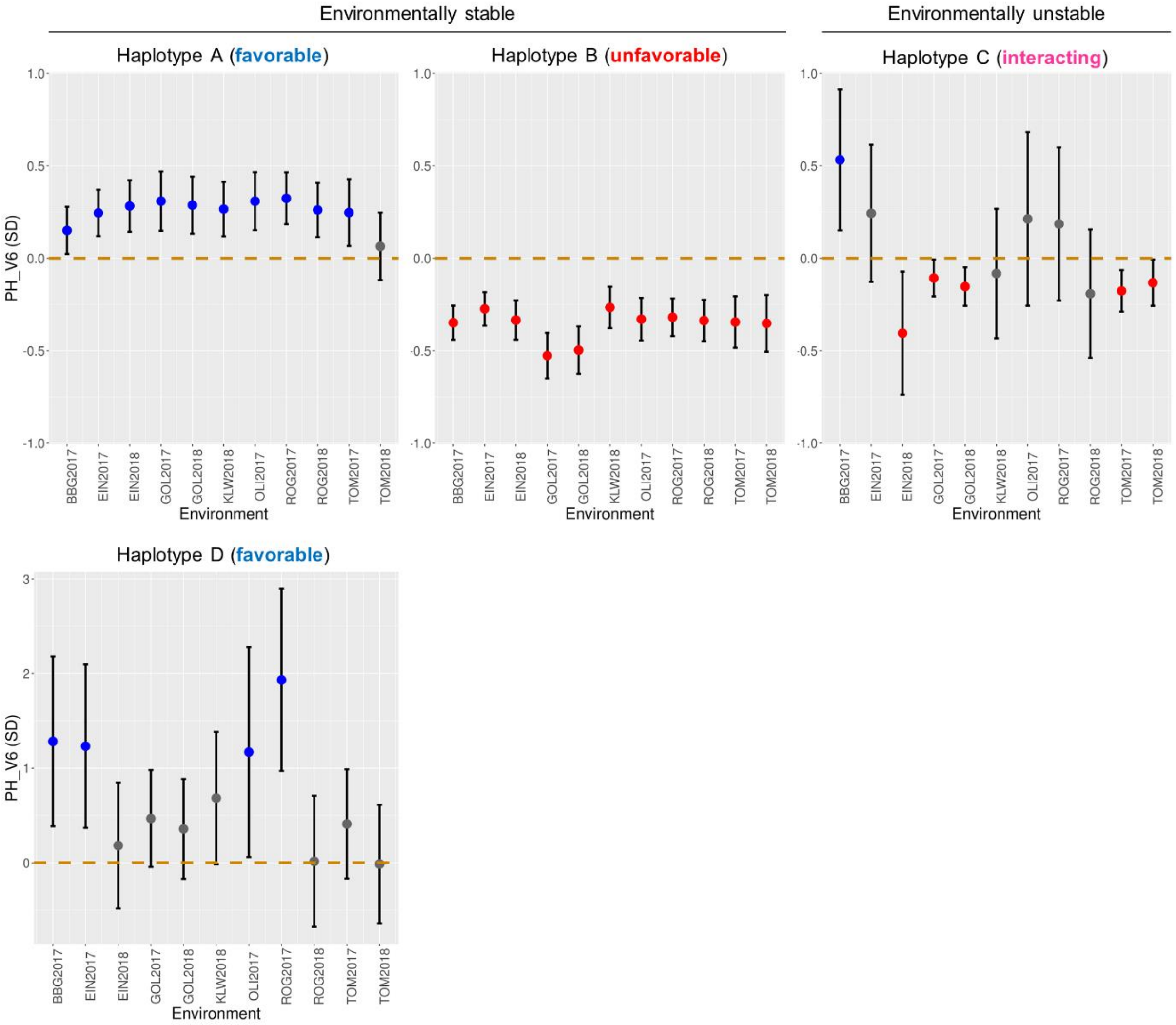

Supplementary Fig. 5: Examples of favorable, unfavorable and interacting haplotypes. Environment-specific effect estimates (mean ± twice the standard error) in units of phenotypic standard deviations for four haplotypes associated with PH_V6 (positive, negative and non-significant effects in blue, red and gray, respectively). Haplotype A on chromosome 3 showed significant positive effects in ten out of eleven environments increasing early plant growth, thus representing an environmentally stable favorable haplotype. Haplotype B on chromosome 10 showed negative effects across all eleven environments, decreasing early plant growth thus categorized as environmentally stable unfavorable haplotype. The effect sign of haplotype C on chromosome 9 varied depending on the environment and thus was classified as interacting. Haplotype D on chromosome 9 represents another example of an environmentally stable favorable haplotype, showing positive effects in all environments where the association was significant. Source data are provided as a Source Data file.

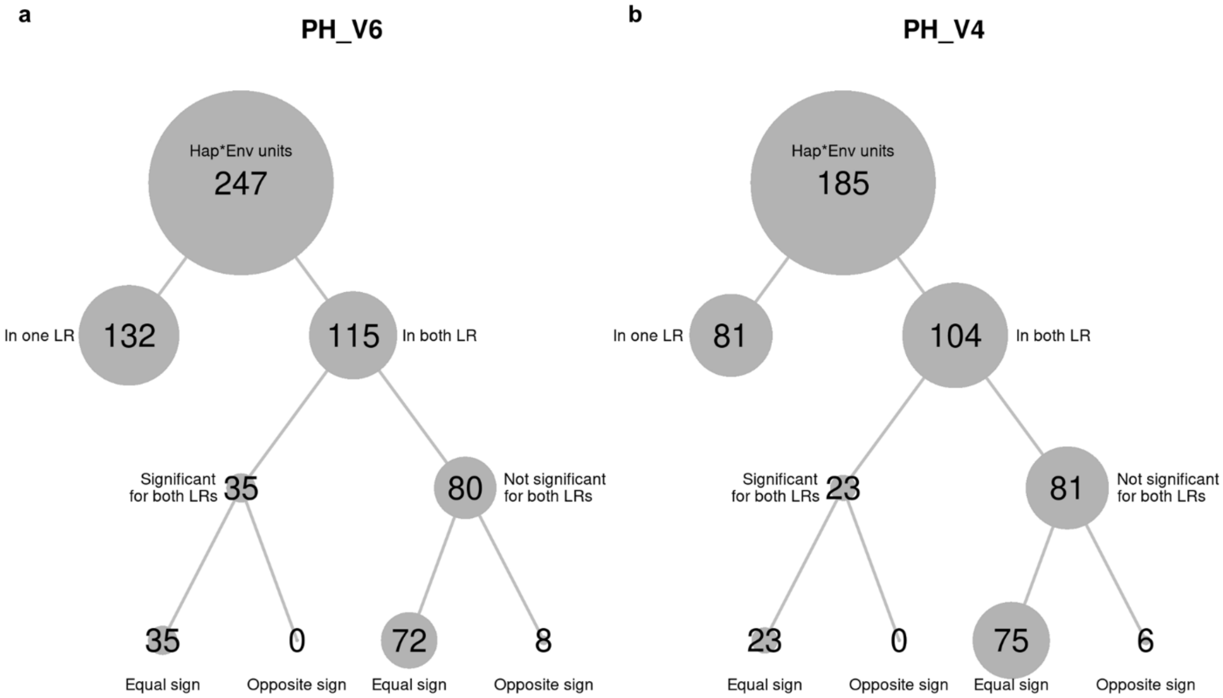

Supplementary Fig. 6: Comparison of haplotype effects between landraces. (a) For PH_V6, 46 of the 48 significantly associated haplotypes were present in at least one of the two landraces KE and PE (two occurred only in LL). The sum of haplotype by environment combinations for which the respective haplotypes were significant was 247. Thereof, 132 environment-specific associations resulted from 27 haplotypes only present in either KE or PE, while 115 associations resulted from 19 haplotypes present in both landraces. Thereof, 35 associations (involving 12 haplotypes) were significant for both landraces, whereas 80 associations were significant for one landrace. All 35 associations significant for both landraces had equal effect signs for both landraces. For the 80 associations only significant for one landrace, 72 had equal effect signs for both landraces. **(b)** Analogous to (a) for PH_V4. Here, 36 haplotypes associated to PH_V4 were present in KE and/or PE. Thereof, 21 haplotypes were present in both KE and PE. The 23 environment-specific associations significant for both landraces involved 13 haplotypes.

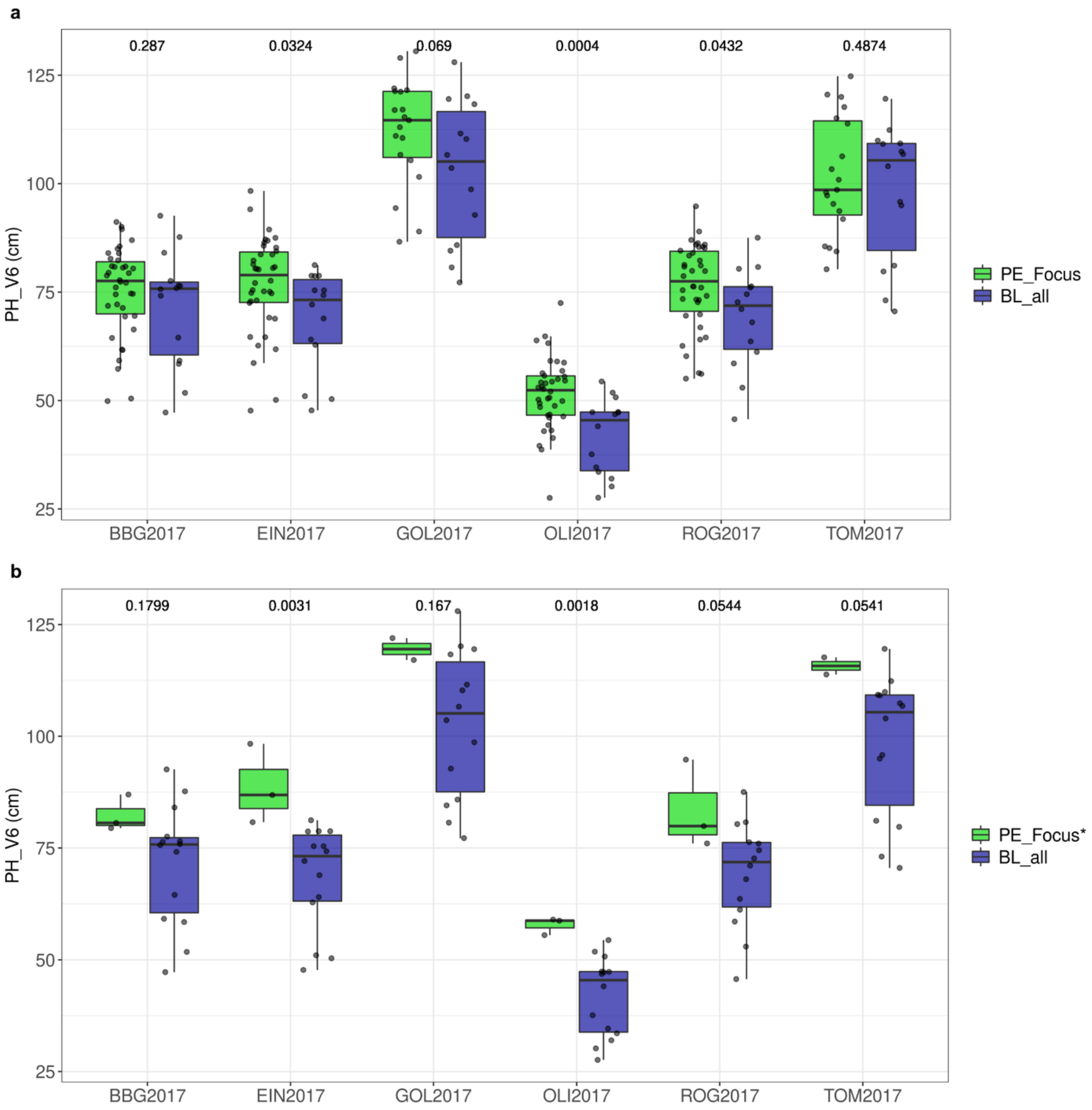

Supplementary Fig. 7: Phenotypic values for PH_V6 in six locations in 2017 for 14 breeding lines (BL_all) and a subset of DH lines derived from the landrace PE (PE_Focus) carrying a focus haplotype. The focus haplotypes in **(a)** and **(b)** refer to haplotype A and D described in Supplementary Fig. 5, respectively. Numbers on the top refer to *P*-values testing the significance of mean differences between BL_all and PE_Focus (permutation test, two-sided). Boxplots show the upper and lower quartile, median (bold horizontal bar) and whiskers (vertical lines). Points outside the whiskers indicate values ±1.5 times the interquartile range. Source data are provided as a Source Data file. *based on three data points only.

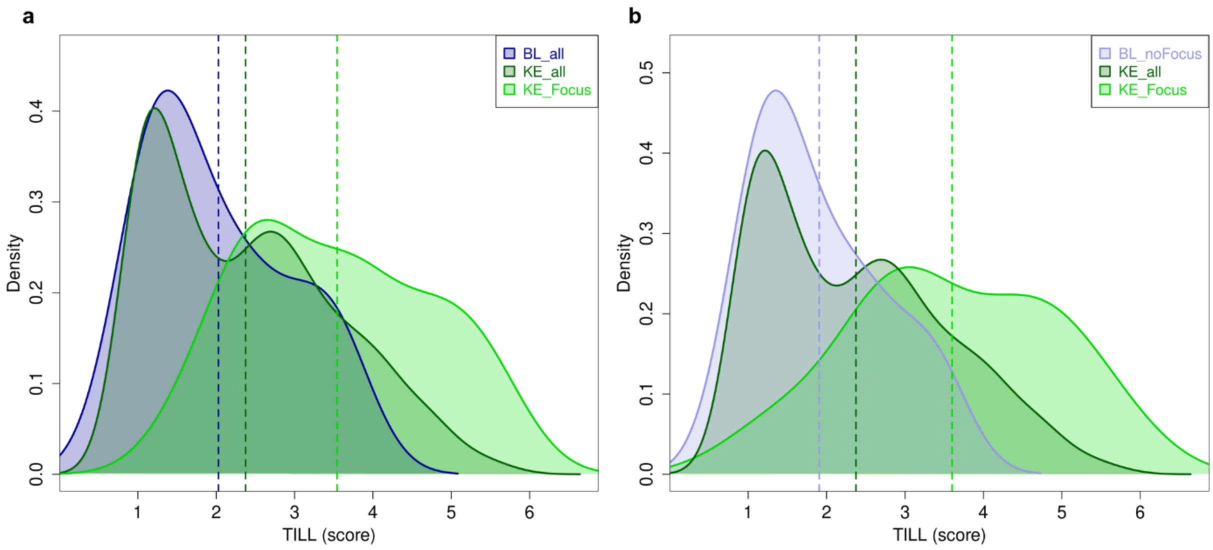

Supplementary Fig. 8: Unfavorable focus haplotypes increasing TILL. Estimated densities of phenotypic values (BLUEs across locations in 2017) for TILL for 14 breeding lines (BL_all), 462 DH lines of landrace KE (KE_all) as well as for DH lines of KE carrying **(a)** a focus haplotype on chromosome 1 (at the *tb1* locus; KE_Focus, 35 lines) and **(b)** a focus haplotype on chromosome 5 (KE_Focus, 16 lines). As one of the 14 breeding lines carried the focus haplotype on chromosome 5, comparisons were made with the remaining 13 lines (BL_noFocus). Vertical lines indicate the mean of each group. The difference in means between BL_all and KE_all was not significant ($P > 0.277$; permutation test, two-sided). Source data are provided as a Source Data file.

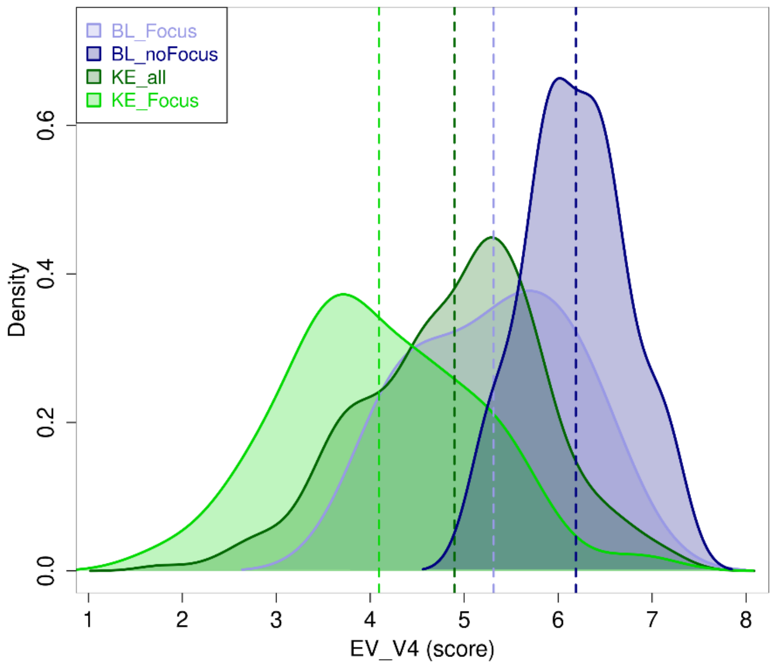

Supplementary Fig. 9: Unfavorable focus haplotype decreasing EV_V4 in landraces and breeding lines. Estimated densities of phenotypic values (BLUEs across locations in 2017) for EV_V4 for 471 DH lines of landrace KE (KE_all) as well as for 49 DH lines of KE (KE_Focus) and six breeding lines (BL_Focus) carrying the focus haplotype on chromosome 1 and eight breeding lines (BL_noFocus) not carrying the respective haplotype. Vertical lines indicate the mean of each group. Source data are provided as a Source Data file.

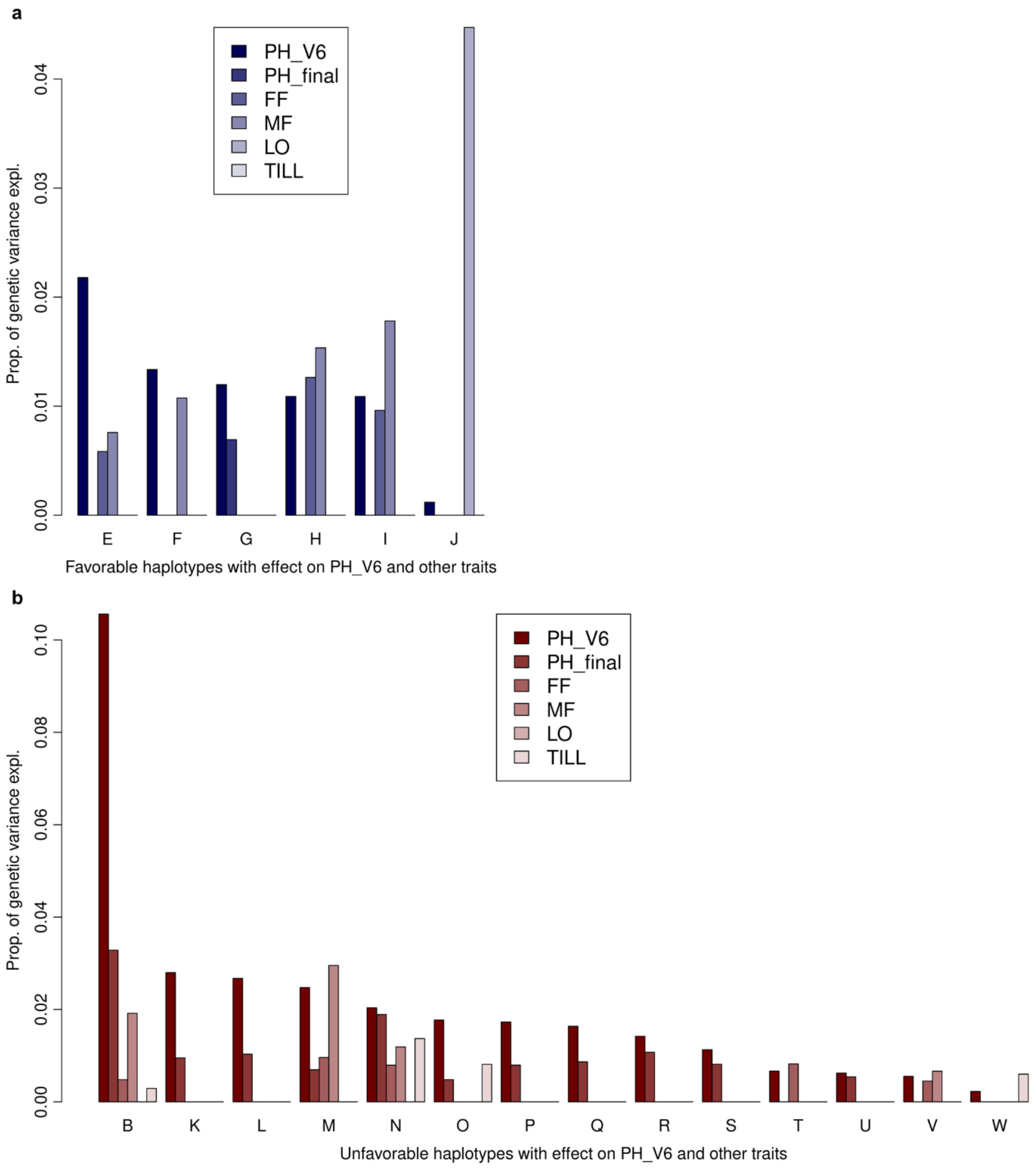

Supplementary Fig. 10: Haplotypes with effects on multiple traits. Proportions of explained genetic variance per trait for each of **(a)** six favorable and **(b)** 14 unfavorable focus haplotypes associated with PH_V6 with significant effects on PH_final, FF, MF, LO, and/or TILL. All haplotypes had equal effect signs for PH_V6 and PH_final/LO/TILL and opposite effect signs for PH_V6 and FF/MF, respectively. Source data are provided as a Source Data file.

Supplementary Tables

Supplementary Table 1: Diversity measures for landrace-derived DH lines and breeding lines. The table lists the number of lines (*n*), polymorphism information content (PIC), gene diversity (*H*) and minimum number of historical recombination events (nR) for DH libraries derived from the three landraces KE, LL and PE as well as for the combined set of DH lines (DH all) and a set of breeding lines (BL). PIC and *H* were calculated based on SNPs (PIC_{SNP} and H_{SNP}) as well as haplotypes (PIC_{hap} and H_{hap}). Haplotypes were constructed and nR calculated for non-overlapping genomic windows of 10 SNPs.

**Source**	***n***	**PIC_{SNP}**	**PIC_{hap}**	**H_{SNP}**	**H_{hap}**	**nR**
KE	501	0.199	0.458	0.249	0.515	0.323
LL	31	0.224	0.562	0.279	0.608	0.674
PE	409	0.187	0.446	0.232	0.499	0.497
DH all	941	0.233	0.562	0.289	0.610	1.187
BL	65	0.254	0.628	0.316	0.668	1.202

Supplementary Table 2: Overview of the phenotypic data analyzed in this study. Lists for each trait the abbreviation, the way of measurement, the growth stages (according to Abendroth et al. 2011) at which measurements were conducted, the number of lines (*n* DHs) for which data was available and the number of environments (*n* Env.) in which the traits were measured.

**Trait**	**Abbr.**	**Measurement**	**Growth Stages**	***n* DHs**	***n* Env.**
Early plant height	PH	Total height in cm, from soil surface to highest tip of upwards stretched leaves, mean of three representative plants per plot	V4, V6	899	11
Early vigor	EV	Score 1-9, visual appearance of whole plot, 1 = very small plants with discolored leaves, 9 = very vigorous and healthy looking plants	V4, V6	899	11
Final plant height	PH_final	Total height in cm, from soil surface to lowest tassel branch, mean of three representative plants per plot	R4	899	11
Female flowering	FF	Days after sowing until 50% of plants show silks	R1	899	10
Male flowering	MF	Days after sowing until 50% of plants shed pollen	R1	899	5
Lodging	LO	Score 1-9, 1 = no lodging, 9 = all plants show severe lodging	R6	869	4
Tillering	TILL	Score 1-9, 1 = no tillers, 9 = many and long tillers	V8-V10	899	5

Supplementary References

1. Abendroth, L.J., Elmore, R.W., Boyer, M.J. & Marlay, S.K. *Corn Growth and Development*, (Iowa State University, University Extension, 2011).
