# Supplementary material for: Discovery of beneficial haplotypes for complex traits in maize landraces
Source: Nat Commun. 2020 Oct 2;11:4954. doi: 10.1038/s41467-020-18683-3 (PMC7532167; doi:10.1038/s41467-020-18683-3)
Supplement: Supplementary file 3 — Reporting Summary [file 41467_2020_18683_MOESM3_ESM.pdf]

nature research

Corresponding author(s): Chris-Carolin Schön

Last updated by author(s): Aug 24, 2020

Reporting Summary

Nature Research wishes to improve the reproducibility of the work that we publish. This form provides structure for consistency and transparency in reporting. For further information on Nature Research policies, see our Editorial Policies and the Editorial Policy Checklist.

Statistics

For all statistical analyses, confirm that the following items are present in the figure legend, table legend, main text, or Methods section.

n/a	Confirmed	
☐	☒	The exact sample size (*n*) for each experimental group/condition, given as a discrete number and unit of measurement
☐	☒	A statement on whether measurements were taken from distinct samples or whether the same sample was measured repeatedly
☐	☒	The statistical test(s) used AND whether they are one- or two-sided<br>*Only common tests should be described solely by name; describe more complex techniques in the Methods section.*
☐	☒	A description of all covariates tested
☐	☒	A description of any assumptions or corrections, such as tests of normality and adjustment for multiple comparisons
☐	☒	A full description of the statistical parameters including central tendency (e.g. means) or other basic estimates (e.g. regression coefficient) AND variation (e.g. standard deviation) or associated estimates of uncertainty (e.g. confidence intervals)
☐	☒	For null hypothesis testing, the test statistic (e.g. *F*, *t*, *r*) with confidence intervals, effect sizes, degrees of freedom and *P* value noted<br>*Give P values as exact values whenever suitable.*
☒	☐	For Bayesian analysis, information on the choice of priors and Markov chain Monte Carlo settings
☐	☒	For hierarchical and complex designs, identification of the appropriate level for tests and full reporting of outcomes
☐	☒	Estimates of effect sizes (e.g. Cohen's *d*, Pearson's *r*), indicating how they were calculated

Our web collection on statistics for biologists contains articles on many of the points above.

Software and code

Policy information about availability of computer code

Data collection	Genotypic data of 2 breeding lines were downloaded from the HapMapV3.2.1 database (https://cbsusrv04.tc.cornell.edu/users/panzea/download.aspx?filegroupid=34)
Data analysis	Genotype calling: Affymetrix Axiom Analysis Suite Software, version 3.0.1.4<br>Imputation: BEAGLE, version 5.0<br>GWAS: GEMMA, version 0.98.1<br><br>All remaining data analyses were performed in 'R' version 3.6.0. The following R packages were used for the different analyses:<br>- 'ape' version 5.3 (for PCoA)<br>- 'asreml' version 3.0 (for fitting mixed linear models)<br>- 'ggplot2' version 3.2.0 (for plotting)<br>- 'plot3D' version 1.3 (for plotting)<br>- 'synbreed' version 0.12-9 (for calculation of the genomic relationship matrices from SNP data)<br>- 'VennDiagram' version 1.6.20 (for plotting)<br>- 'zoo' version 1.8-6 (for sliding windows for haplotype construction)<br><br>All costum code is available at https://github.com/Manfred-Mayer/GWAS_DHs_landraces

For manuscripts utilizing custom algorithms or software that are central to the research but not yet described in published literature, software must be made available to editors and reviewers. We strongly encourage code deposition in a community repository (e.g. GitHub). See the Nature Research guidelines for submitting code & software for further information.

Data

Policy information about availability of data

All manuscripts must include a data availability statement. This statement should provide the following information, where applicable:

- Accession codes, unique identifiers, or web links for publicly available datasets
- A list of figures that have associated raw data
- A description of any restrictions on data availability

The genotypic data of 941 DH lines and the phenotypic data of 899 DH lines and 14 breeding lines are available in figshare with the identifier https://doi.org/10.6084/m9.figshare.12137142. The 600k data of 63 breeding lines can be accessed at https://dx.doi.org/10.6084/m9.figshare.3427040.v1, while for two lines genotypic data based on whole genome sequences were downloaded from CyVerse Data Store (http://cbsusrv04.tc.cornell.edu/users/panzea/download.aspx?filegroupid=34). Source data are provided with this paper.

Field-specific reporting

Please select the one below that is the best fit for your research. If you are not sure, read the appropriate sections before making your selection.

[x] Life sciences [ ] Behavioural & social sciences [ ] Ecological, evolutionary & environmental sciences

For a reference copy of the document with all sections, see nature.com/documents/nr-reporting-summary-flat.pdf

Life sciences study design

All studies must disclose on these points even when the disclosure is negative.

Sample size	Sample size for GWAS was 899 lines derived from three landraces (after quality filtering). Generating DH lines from landraces is challenging compared to breeding material. The extensive phenotypic evaluation is labor and time consuming. The 899 lines were the maximum number for which the generation of high quality data was feasable within the scope of the project. Compared to the literature, sample sizes of multiple hundred individuals can be considered as large and sufficient for association studies. Also our results show that the size was sufficient for successfully mapping significant trait associations.
Data exclusions	All filtering steps for DH lines have been described in detail by Hölker et al. 2019 (https://doi.org/10.1007/s00122-019-03428-8). No additional data were excluded from the study.
Replication	For the field evaluations, each DH line was replicated twice in each of the eleven environments. Heritabilities and repeatabilities were high (Hölker et al. 2019; https://doi.org/10.1007/s00122-019-03428-8).
Randomization	All genotypes were randomly allocated into experimental units.
Blinding	Experimental units were associated with plot numbers only and not with genotype names, i.e. investigators were blinded to group allocation during data collection.

Reporting for specific materials, systems and methods

We require information from authors about some types of materials, experimental systems and methods used in many studies. Here, indicate whether each material, system or method listed is relevant to your study. If you are not sure if a list item applies to your research, read the appropriate section before selecting a response.

Materials & experimental systems

n/a	Involved in the study
[x]	[ ] Antibodies
[x]	[ ] Eukaryotic cell lines
[x]	[ ] Palaeontology and archaeology
[x]	[ ] Animals and other organisms
[x]	[ ] Human research participants
[x]	[ ] Clinical data
[x]	[ ] Dual use research of concern

Methods

n/a	Involved in the study
[x]	[ ] ChIP-seq
[x]	[ ] Flow cytometry
[x]	[ ] MRI-based neuroimaging
