# Supplementary material for: Discovery of beneficial haplotypes for complex traits in maize landraces
Source: Nat Commun. 2020 Oct 2;11:4954. doi: 10.1038/s41467-020-18683-3 (PMC7532167; doi:10.1038/s41467-020-18683-3)
Supplement: Supplementary file 4 — Description of Additional Supplementary Files [file 41467_2020_18683_MOESM4_ESM.pdf]

Description of Additional Supplementary Files

File name: Supplementary Data 1
Description: List of identified trait associations. Lists for each trait the position (chromosome, start and end) and size (kb) of each identified trait-associated genomic region as well as the start and end positions of the selected focus haplotype. Further, the range of the corresponding environment-specific haplotype effects (in units of environment-specific standard deviations) together with the respective environments in which the minimum and maximum effects were observed are shown. The table also states for each region the number of environments in which the focus haplotype had a significant effect. The number of annotated genes within each region (including segments 5 kb upstream of genes) according to the B73 AGPv4 reference sequence are shown.
