# Supplementary material for: Discovery of beneficial haplotypes for complex traits in maize landraces
Source: Nat Commun. 2020 Oct 2;11:4954. doi: 10.1038/s41467-020-18683-3 (PMC7532167; doi:10.1038/s41467-020-18683-3)
Supplement: Supplementary file 2 — Peer Review [file 41467_2020_18683_MOESM2_ESM.pdf]

Reviewer #1 (Remarks to the Author):

This is a very important research based on sound materials and methods and appropriate data handlings and analysis. The findings are well written considering the available literature relevant to this research.

There are however some minor edit suggestions to improve the flow and readability, most of which relate to using a phrasing in the Results section that belongs to the Materials and Methods section. They are in line 69-74, 80-81, 94-96, 137-138, and 170-172. The author should delete them, rephrase considering they belong to the Results section or merged with next sentence of true results but avoiding having a long sentence.

The discussion section has also a similar problem as noted for the Results section in line 285. Authors should be able to fixed by rewriting it along with next sentence.

Line 266: It is poor English writing to use "and/or". Authors should be able to distinguish what of the two words is more appropriate for their sentence.

Materials and methods. The authors should provide briefly details of the field experiments and not just using a reference from other experiment in which the same materials were used (e.g. lines 360-361 for 958 doubled haploid lines, and lines 364-365 for 65 checks). A manuscript should be self-contained when describing important methods used for trials.

Reviewer #2 (Remarks to the Author):

The authors report a novel research about how genetic variation in maize landraces can be explored and utilized for crop breeding, which is interesting to others in the community and the wider field. Satistical analysis used is appropriate and valid. The conclusions are original but some further evidence would be required to strengthen them. The paper can be accepted after a major revision by following the comments and suggestions.

Major issues

1. Selection in DH processing: is there any evaluation and potential effects on preferential regeneration of non-landrace genotypes and alleles in DH generation procedure.

2. I wonder where the heterozygous genotypes come from as the DH lines were doubled haploids (see lines 342-343). First, if heterozygous loci exist, it would be interesting to know what have contributed to the generation of heterozygosity. Second, that "all missing values were imputed separately for each landrace" will replace the real heterozygous loci by imputed homozygous loci. Therefore, it is better to know if there is any heterozygous loci and if yes, how it happened.

3. Favorable haplotypes from both the landrace and breeding panels should be investigated and reported. With a long term of selection and accumulation of favorable mutations, breeding lines may host some favorable haplotypes that are absent in the landrace lines. Favorable haplotypes in breeding lines, particularly those for high yielding and plant-density tolerance, could be introgressed for improvement of landrace lines. Therefore, it would be interesting to know how many cases elite breeding lines could provide favorable haplotypes. Considering that the landrace lines were derived from only three different landraces while the breeding lines represent a set of lines developed through a long term breeding programs across countries, the two panels should have their unique or specific favorable alleles/haplotypes for many traits.

Overall, 26.2% of the haplotypes of the landrace panel were not present in the breeding lines (lines

86-87), while the landrace panel captured 72.4% of the haplotypes present in the panel of breeding lines (lines 91-92), indicating that the breeding lines hosted more haplotypes than the landrace panel. As more lines are included in the landrace panel, the landrace panel should have had more haplotypes if it has the same level of genetic diversity as the breeding line panel.

Six favorable focus haplotypes (11%) were absent in the set of breeding lines representing potential novel variation for elite germplasm improvement (lines 162-164). How many favorable focus haplotypes were present in breeding lines but absent in the landrace DH lines?

A more careful and comprehensive comparison between the landrace lines and the breeding lines should be provided. A full set of contracting analysis should be done by using the breeding lines as genetic variation source for improvement of landraces.

4. To understand better the level of genetic diversity in two germplasm panels, diversity analysis (PIC and other gene diversity criteria) should be done based on both SNPs and haplotypes.

5. Haplotype-based GWAS should be compared with SNP-based GWAS. Manhattan plots for GWAS should be provided for some traits, for example, TILL involving tb1 locus.

6. I have a major concern about the section "Linking novel haplotype variation to phenotypes", where only some genomic regions were discussed as examples by comparison with only 14 breeding lines and if understand right, in some cases the 14 lines were further classified into two subgroups based on the focus haplotypes, resulting in each with a limited population size for statistical tests.

Minor issues:

Title: Add the word "maize" to the title.

Abstract: (1) Not sure what is "pre-selected based on molecular and phenotypic information" according to the sentence per se. (2) Indicate that all the DH and breeding lines were genotyped with the 600k array.

Line 333: replace "based on" by "selected from".

Line 347: Regarding "all alleles were coded according to the B73 AGPv446 forward strand", the two breeding lines, EZ5 and F64, which have SNPs extracted from their whole genome sequencing can be used as controls to check for the coding accuracy by their known relationship with other breeding lines.

Line 379: In "yielding genotype scores 0 and 2 for DH and breeding lines", readers may be not sure which is 0 and which is 2, DH and breeding lines, or presence and absence until they read the more description in the following section.

Lines 395-396: Regarding "a three-dimensional vector of fixed effects (intercept and landrace effects of KE and LL)", why and how the effects of these two landraces (not the two with larger population size) were selected as fixed effects? How this selection could have contributed to all the relevant results?

Line 441: not sure if the term "the respective other trait" is a right description. Please check.

Fig 1b: No significantly different haplotype frequencies could be seen between the landrace lines and breeding lines, as shown by the haplotype numbers and their presence and absence between the two panels. It seems no significant difference in genetic variation was found between the two panels, which is unexpected.

Distribution of favorable haplotypes across the genome may be provided with two different colors, one for those from the landraces and the other from the breeding lines.

Reviewer #3 (Remarks to the Author):

This is nice work describing the analysis of haplotype variation in DH lines extracted from three European landraces and their effects on agronomic traits. The authors demonstrate the identification of a number of unique haplotypes that have favorable effects on some traits like early growth under cold conditions and without unfavorable effects. I've worked in the area of germplasm improvement in maize for some time and we have operated under the assumption that such haplotypes exist, but this paper is one of the few direct demonstrations of this. The complexity of haplotype structure in maize, the masking effects of lack of adaptation, and the generally small effects of novel variants have all hindered this kind of study. The authors mostly eliminated the issues with adaptation by focusing on pre-adapted landraces, and they used sufficiently large sample sizes to get reliable estimates of the haplotype effects. Their approach simplifies the analysis of haplotypes by arbitrarily using 10-SNP windows. Probably this could be improved somehow, but as the authors note, determining the optimal approach to define haplotypes is very difficult, so their approach appears to have been good enough to work for their purposes.

I have little to criticize. The only additional information that would be useful to add, if possible, would be a comparison of the haplotype GWAS and allele frequency estimates to what would have been learned from single-SNP GWAS. The fact that they don't even mention single SNP GWAS or frequencies makes me wonder if they tried that and it did not work very well...or maybe they just guessed it wasn't going to work so they jumped right to the haplotype-based analyses. I would be interested to know if they tried single SNP analyses, and if they found some different results, to give the reader some interpretation on why that is, and to recommend approaches going forward. They could add the individual SNP frequency spectra to Figure 4 (maybe in a separate panel).

Jim Holland

Note:

All answers are written in blue italics. All line numbers refer to the revised version of the manuscript with (non-hidden) track changes.

Reviewer #1 (Remarks to the Author):

This is a very important research based on sound materials and methods and appropriate data handlings and analysis. The findings are well written considering the available literature relevant to this research.

There are however some minor edit suggestions to improve the flow and readability, most of which relate to using a phrasing in the Results section that belongs to the Materials and Methods section. They are in line 69-74, 80-81, 94-96, 137-138, and 170-172. The author should delete them, rephrase considering they belong to the Results section or merged with next sentence of true results but avoiding having a long sentence.

We changed these sentences as suggested (lines 71-80, 92-95, 106-109, 154-157 and 188-192).

The discussion section has also a similar problem as noted for the Results section in line 285. Authors should be able to fixed by rewriting it along with next sentence.

We changed the sentence as suggested (lines 305-307).

Line 266: It is poor English writing to use "and/or". Authors should be able to distinguish what of the two words is more appropriate for their sentence.

We agree that "and/or" is not precise and actually in this case the combination of both moderate effect sizes <u>and</u> comparably low frequency is decisive, therefore we changed it to only "and" (line 286).

Materials and methods. The authors should provide briefly details of the field experiments and not just using a reference from other experiment in which the same materials were used (e.g. lines 360-361 for 958 doubled haploid lines, and lines 364-365 for 65 checks). A manuscript should be self-contained when describing important methods used for trials.

We included a brief description of the experimental set up in the manuscript (lines 382-391).

Reviewer #2 (Remarks to the Author):

The authors report a novel research about how genetic variation in maize landraces can be explored and utilized for crop breeding, which is interesting to others in the community and the wider field. Satistical analysis used is appropriate and valid. The conclusions are original but some further evidence would be required to strengthen them. The paper can be accepted after a major revision by following the comments and suggestions.

Major issues

1. Selection in DH processing: is there any evaluation and potential effects on preferential regeneration of non-landrace genotypes and alleles in DH generation procedure

Based on comparisons between original landrace individuals and derived DH lines we found that "The DH libraries ... represented their respective landraces accurately in terms of molecular variance." (Hölker et al. 2019). When analyzing the molecular relationship between individuals, e.g. by principal coordinate analysis (Hölker et al. 2019), every DH line clearly clustered together with the original individuals from the same landrace.

DH lines were produced directly from the landraces using the in vivo haploid induction method (Röber et al. 2005). No further crossing with any non-landrace line was conducted. If the reviewer is concerned about potential inclusion of the inducer genotype, we refer to the quality control filter for <5% heterozygous sites per line, which eliminated lines resulting from potentially wrongly selected diploid kernels.

Röber FK, Gordillo GA, Geiger HH (2005) In vivo haploid induction in maize-performance of new inducers and significance of doubled haploid lines in hybrid breeding. Maydica 50:275

2. I wonder where the heterozygous genotypes come from as the DH lines were doubled haploids (see lines 342-343). First, if heterozygous loci exist, it would be interesting to know what have contributed to the generation of heterozygosity. Second, that "all missing values were imputed separately for each landrace" will replace the real heterozygous loci by imputed homozygous loci. Therefore, it is better to know if there is any heterozygous loci and if yes, how it happened.

The proportion of heterozygous calls was very low (0.19% of the genotype matrix). Further, the heterozygous calls did not cluster in any specific genomic region but were randomly scattered across the genome. They can therefore be expected to result from technical errors in the genotyping process. As with any genotyping method, it is also common in SNP arrays that a small percentage of genotype calls is erroneous. We added the proportion of calls indicating heterozygosity for both the DH as well as the breeding lines and a short statement that they are technical artefacts in the text (lines 362-363 and 373).

3. Favorable haplotypes from both the landrace and breeding panels should be investigated and reported. With a long term of selection and accumulation of favorable mutations, breeding lines may host some favorable haplotypes that are absent in the landrace lines. Favorable haplotypes in breeding lines, particularly those for high yielding and plant-density tolerance, could be introgressed for improvement of landrace lines. Therefore, it would be interesting to know how many cases elite breeding lines could provide favorable haplotypes. Considering that the landrace lines were derived from only three different landraces while the breeding lines represent a set of lines developed through a long term breeding programs across countries, the two panels should have their unique or specific favorable alleles/haplotypes for many traits.

We agree that it is very likely that breeding lines harbor favorable haplotypes absent in our panel of landrace derived lines. Our results in fact indicate that both panels have a considerable proportion of haplotypes which are absent in the respective other panel (lines 94-104). Our objective was to discover novel variation by haplotype mining of landraces for broadening the narrow genetic base of the current breeding germplasm and use this information to improve elite lines. The reviewer had apparently the opposite direction in mind, i.e., improvement of DH lines from landraces by introgression of alleles from elite lines. However, this was not the goal of the current study. As reviewer #3 confirmed, the detection of favorable haplotypes not confounded with adaptation related traits in landraces is very difficult and requires huge efforts

by genotyping and phenotyping large panels of lines. Doing the same for breeding material was beyond the scope of this study.

Overall, 26.2% of the haplotypes of the landrace panel were not present in the breeding lines (lines 86-87), while the landrace panel captured 72.4% of the haplotypes present in the panel of breeding lines (lines 91-92), indicating that the breeding lines hosted more haplotypes than the landrace panel. As more lines are included in the landrace panel, the landrace panel should have had more haplotypes if it has the same level of genetic diversity as the breeding line panel.

Adding the diversity parameters (see statement 4) was very helpful and assists in clarification here. We agree that the number of haplotypes per panel should be a function of the number of lines investigated. However, as indicated now in lines 88-90, "the (three) landraces represent self-contained populations whereas the breeding line panel was derived from a large number of different source populations across Europe" (Unterseer et al. 2016). Therefore, it cannot be determined a priori how many haplotypes to expect solely by the number of lines in each of the two panels. It can only be expected that the three landraces capture only part of the allelic diversity of European flint germplasm (Mayer et al., 2017).

Six favorable focus haplotypes (11%) were absent in the set of breeding lines representing potential novel variation for elite germplasm improvement (lines 162-164). How many favorable focus haplotypes were present in breeding lines but absent in the landrace DH lines?

A more careful and comprehensive comparison between the landrace lines and the breeding lines should be provided. A full set of contracting analysis should be done by using the breeding lines as genetic variation source for improvement of landraces.

We had to prioritize in the choice of material for phenotyping and genotyping. As our goal was to improve breeding material by taking the landraces as genetic variation source, we concentrated most of the available resources on generating, genotyping and phenotyping landrace derived DH lines. An independent identification of favorable haplotypes in breeding lines would have required a much larger body of data.

4. To understand better the level of genetic diversity in two germplasm panels, diversity analysis (PIC and other gene diversity criteria) should be done based on both SNPs and haplotypes

Thank you very much for this suggestion. We now calculated polymorphism information content (PIC) as well as gene diversity (H) based on both SNPs and haplotypes for the landrace derived DH lines as well as for the breeding lines. Further we show the values for the minimum number of recombination events (nR) for the different germplasm groups. The values are listed in the new Suppl. Table 1 and a short description was added in the Results (lines 84-91) and Methods (lines 413-420) sections.

5. Haplotype-based GWAS should be compared with SNP-based GWAS. Manhattan plots for GWAS should be provided for some traits, for example, TILL involving tb1 locus.

Following this suggestion, we included Manhattan plots exemplarily for TILL for both SNP based and haplotype based GWAS in the manuscript (lines 108-113; 467-468; new Suppl. Fig. 2). In general, SNP based GWAS lead to the identification of very similar regions as haplotype based GWAS. However, as we also stated in the manuscript (lines 111-113), the comparison of alleles between populations (like landraces and elite material) is more meaningful with haplotypes. Therefore, we focused on the haplotype-based approach.

6. I have a major concern about the section "Linking novel haplotype variation to phenotypes", where only some genomic regions were discussed as examples by comparison with only 14 breeding lines and if understand right, in some cases the 14 lines were further classified into two subgroups based on the focus haplotypes, resulting in each with a limited population size for statistical tests.

We compared the phenotypic performance between landrace derived DH lines and a subset of 14 breeding lines, which were chosen to represent the diversity spectrum of the panel of 65 breeding lines. P-values for the conducted statistical tests as well as the respective sample sizes are given in the text. As we were testing the effects of favorable haplotypes not present in the breeding lines, the contrast was generally based on the 14 chosen breeding lines. For one of the unfavorable haplotypes we felt it was useful to show its effect within the breeding lines (lines 232-234). We agree that sample size is small, but the statistical test was significant. As pointed out in the discussion section (lines 309-311), validation of the haplotype effects found in landraces with respect to improvement of a given set of breeding lines will always have to come from crosses of landrace derived material with the specific elite material one aims to improve.

Minor issues:

Title: Add the word "maize" to the title.

Changed.

Abstract: (1) Not sure what is "pre-selected based on molecular and phenotypic information" according to the sentence per se. (2) Indicate that all the DH and breeding lines were genotyped with the 600k array.

(1) We rephrased the sentence to be more specific (lines 17-18). In the main text we describe, that landraces were pre-selected for adaptation to target environments (lines 58-60) as well as for phenotypic variation in cold-related traits assessed in field trials and for population genetic analyses (lines 351-352; also lines 114-115 and 281-283).

(2) Changed (lines 17-22).

Line 333: replace "based on" by "selected from".

Changed (line 353).

Line 347: Regarding "all alleles were coded according to the B73 AGPv446 forward strand", the two breeding lines, EZ5 and F64, which have SNPs extracted from their whole genome sequencing can be used as controls to check for the coding accuracy by their known relationship with other breeding lines.

Maybe this was a misunderstanding, if the question is about the general data quality (i.e. correctness of genotype calls) usually array data perform better than sequence data. In fact, for studies, where multiple data sources are available, generally array data is considered as the reference for determining the quality of sequence data (e.g. Kishikawa et al. 2019; personal communication Ed Buckler – Cornell University). In our case, all lines, except EZ5 and F64, were genotyped with the 600k array.

If the concern is about the forward/reverse strand coding: While the HapMap sequence data is according to the B73v4 forward strand, the alleles of the array were initially partly based on the forward and partly based on the reverse strand. We coded all markers according to the

B73v4 forward strand based on the orientation of the respective oligo-nucleotides when mapping them on the reference genome. For 14 lines (but not for EZ5 and F64) we had 600k array data as well as whole genome sequence (HapMap) data available. For these 14 lines we compared the coding at overlapping sites (424,400) between the two data sources and found very high congruence (on average 99.45% identical; median 99.72%).

Kishikawa T, Momozawa Y, Ozeki T, et al (2019) Empirical evaluation of variant calling accuracy using ultra-deep whole-genome sequencing data. Sci Rep 9:1784. https://doi.org/10.1038/s41598-018-38346-0

Line 379: In "yielding genotype scores 0 and 2 for DH and breeding lines", readers may be not sure which is 0 and which is 2, DH and breeding lines, or presence and absence until they read the more description in the following section.

We agree that this phrasing can be confusing. We deleted "for DH and breeding lines" (line 410) as it should be clear, that it was applied to all lines and that all considered lines are fully homozygous.

Lines 395-396: Regarding "a three-dimensional vector of fixed effects (intercept and landrace effects of KE and LL)", why and how the effects of these two landraces (not the two with larger population size) were selected as fixed effects? How this selection could have contributed to all the relevant results?

The way we coded it, PE represents the reference (intercept) and the landrace effects of KE and LL refer to the deviation of the means of these landraces from the mean of PE. If a common intercept and effects for all three landraces were included in the model, estimability of the parameters is only warranted with a restriction. The selection of which landrace is used as reference has no effect on the outcome of the analysis.

Line 441: not sure if the term "the respective other trait" is a right description. Please check.

We agree that this is a somewhat odd wording. We changed it to just "other traits" (lines 481-482).

Fig 1b: No significantly different haplotype frequencies could be seen between the landrace lines and breeding lines, as shown by the haplotype numbers and their presence and absence between the two panels. It seems no significant difference in genetic variation was found between the two panels, which is unexpected.

As we wrote in lines 94-97 and 277-282, similarities in haplotype frequencies can be expected if material shares historical ancestry. Both, breeding lines and the selected landraces represent the European flint germplasm and thus are adapted to similar environmental conditions and are expected to share historical ancestors. Nevertheless, despite similar patterns of haplotype frequencies, the proportion of haplotypes overlapping between the two panels was only about 72% (BLs) to 74% (DHs), thus both panels carried haplotypes absent in the respective other panel (lines 94-104).

Distribution of favorable haplotypes across the genome may be provided with two different colors, one for those from the landraces and the other from the breeding lines.

GWAS and thus the detection of favorable haplotypes in the breeding lines was not feasible in this study, as indicated above. The positions of the haplotypes with favorable/unfavorable effects detected in the landraces are plotted exemplarily for PH_V6 in Fig 3a.

Reviewer #3 (Remarks to the Author):

This is nice work describing the analysis of haplotype variation in DH lines extracted from three European landraces and their effects on agronomic traits. The authors demonstrate the identification of a number of unique haplotypes that have favorable effects on some traits like early growth under cold conditions and without unfavorable effects. I've worked in the area of germplasm improvement in maize for some time and we have operated under the assumption that such haplotypes exist, but this paper is one of the few direct demonstrations of this. The complexity of haplotype structure in maize, the masking effects of lack of adaptation, and the generally small effects of novel variants have all hindered this kind of study. The authors mostly eliminated the issues with adaptation by focusing on pre-adapted landraces, and they used sufficiently large sample sizes to get reliable estimates of the haplotype effects. Their approach simplifies the analysis of haplotypes by arbitrarily using 10-SNP windows. Probably this could be improved somehow, but as the authors note, determining the optimal approach to define haplotypes is very difficult, so their approach appears to have been good enough to work for their purposes.

I have little to criticize. The only additional information that would be useful to add, if possible, would be a comparison of the haplotype GWAS and allele frequency estimates to what would have been learned from single-SNP GWAS. The fact that they don't even mention single SNP GWAS or frequencies makes me wonder if they tried that and it did not work very well...or maybe they just guessed it wasn't going to work so they jumped right to the haplotype-based analyses. I would be interested to know if they tried single SNP analyses, and if they found some different results, to give the reader some interpretation on why that is, and to recommend approaches going forward. They could add the individual SNP frequency spectra to Figure 4 (maybe in a separate panel).

During the course of our analyses we had performed single-SNP based GWAS and compared it with our haplotype based approach. The identified genomic regions were very similar. As our focus lied on the identification of haplotypes, we decided to exclude the SNP based GWAS from the manuscript to make the main text more concise. Of course, the reviewer is right that it is relevant for the topic. Therefore, we included a small section in the results (lines 108-113) and methods (lines 467-468), as well as Manhattan plots comparing haplotype and SNP based results (new Suppl. Figure 2) exemplarily for TILL, as suggested by reviewer #2.

Additional adjustments:

In addition to the suggestions of the reviewers, we made the following corrections:

We included Eva Bauer as co-author as she was involved in data generation. Accordingly, we added her also in the author contributions section.

Fig2: The number of detected trait associated genomic regions for LO was corrected from 50 to 51. The Table of regions (Supplementary Table 2) actually has 51 entries for LO, but in Fig2 only 50 were listed.

We updated the number of haplotypes used for GWAS from 153,730 to 154,104 (line 424). Also the values for the average window sizes and average *nR* and *HH* changed slightly (lines 171-172 and 407-408). In the earlier version, values had erroneously been extracted from an older analysis. This had no effect on the general outcome and conclusions.

REVIEWERS' COMMENTS:

Reviewer #2 (Remarks to the Author):

A great job done to revise the manuscript and respond to reviewers' comments. I have no further comments for the authors.

Reviewer #3 (Remarks to the Author):

Authors have responded to all previous review comments adequately. This is nice work, congratulations. Jim Holland

Note:

All answers are written in blue italics.

Reviewer #2 (Remarks to the Author):

A great job done to revise the manuscript and respond to reviewers' comments. I have no further comments for the authors.

We thank Reviewer #2 for the time of reviewing our manuscript and the positive feedback.

Reviewer #3 (Remarks to the Author):

Authors have responded to all previous review comments adequately. This is nice work, congratulations. Jim Holland.

We thank Jim Holland for the time of reviewing our manuscript and the positive feedback.
